# Metacontrol and body ownership: divergent thinking increases the virtual hand illusion

**DOI:** 10.1007/s00426-018-0976-9

**Published:** 2018-01-10

**Authors:** Ke Ma, Bernhard Hommel

**Affiliations:** 1 Key Laboratory of Personality and Cognition, Faculty of Psychological Science, Southwest University, Beibei, Chongqing, China; 2 Cognitive Psychology Unit, Institute for Psychological Research and Leiden Institute for Brain and Cognition, Leiden University, Wassenaarseweg 52, 2333 AK Leiden, The Netherlands

## Abstract

The virtual hand illusion (VHI) paradigm demonstrates that people tend to perceive agency and bodily ownership for a virtual hand that moves in synchrony with their own movements. Given that this kind of effect can be taken to reflect self–other integration (i.e., the integration of some external, novel event into the representation of oneself), and given that self–other integration has been previously shown to be affected by metacontrol states (biases of information processing towards persistence/selectivity or flexibility/integration), we tested whether the VHI varies in size depending on the metacontrol bias. Persistence and flexibility biases were induced by having participants carry out a convergent thinking (Remote Associates) task or divergent-thinking (Alternate Uses) task, respectively, while experiencing a virtual hand moving synchronously or asynchronously with their real hand. Synchrony-induced agency and ownership effects were more pronounced in the context of divergent thinking than in the context of convergent thinking, suggesting that a metacontrol bias towards flexibility promotes self–other integration.

## Introduction

People are thought to store representations of their own body, so they can recognize themselves and discriminate themselves from others (Gallagher, 2000; Jeannerod, 2003). Popular methods to investigate how people represent their own and other bodies are paradigms producing the rubber hand illusion (RHI; Botvinick & Cohen, 1998) and the virtual hand illusion (VHI; Slater, Perez-Marcos, Ehrsson, & Sanchez-Vives, 2008; Sanchez-Vives et al., 2010). In the original RHI paradigm, participants are facing a rubber hand lying beside/on top of their hidden real hand. If real and rubber hand are stroked synchronously (compared to a condition in which stroking is asynchronous), participants tend to attribute the felt stroking of their real hand to the rubber hand, judge the positions of their real hand as drifting towards the rubber hand and misperceive the rubber hand as their own. In the original VHI paradigm, participants are wearing a data glove on their real hand, which operates a virtual hand on a screen or virtual space in front of them. If virtual and real hand move in synchrony (compared to moving asynchronously), participants tend to perceive a sense of controlling the virtual hand, and misperceive the virtual hand as part of their own body. Hence, tight correlations between multisensory stimuli applied to, or produced by one’s own effector and an artificial effector close to one’s body seem sufficient to incorporate an artificial effector into one’s body representation.

The importance of such bottom-up factors notwithstanding, the aim of the present study was to test whether the ease or degree of incorporating an artificial effector into one’s own body representation depends on cognitive control. Two basic assumptions motivated our approach. First, we assumed that people represent different parts of their body the same way as they represent body-unrelated objects. This assumption is based on the Theory of Event Coding (TEC, Hommel, Müsseler, Aschersleben, & Prinz, 2001; Hommel, 2004), according to which the cognitive system represents both perceived and produced events by integrated networks of codes of the features of these events. Importantly, TEC does not discriminate between social and non-social or self-related and self-unrelated events (Hommel, Colzato, & van den Wildenberg, 2009), and it allows for various degrees of event integration, including the integration of self and other (Colzato, Zech, Hommel, Verdonschot, van den Wildenberg, Hsieh, 2012). Second, we assumed that the degree of event integration depends on the present metacontrol state. According to the metacontrol state model (MSM; Hommel, 2015), this state varies between the poles of extreme persistence (a state characterized by a strong top-down influence of the current action goal, high selectivity, and strong mutual competition between alternative representations, e.g., in decision-making) and extreme flexibility (a state characterized by weak top-down influence of the action goal, strong integration, and weak mutual competition). A bias towards persistence would be associated with the tendency to discriminate between given events whereas a bias towards flexibility would be associated with the tendency to integrate them. Importantly, this holds for both non-social and social events, including people and body parts (cf., Hommel & Colzato, 2017).

Applying these two assumptions to VHI, the paradigm we were using in the present study, motivates the prediction that a bias towards persistence would reduce, and a bias towards flexibility would increase the VHI: given that the illusion implies the integration of the representation of an artificial effector and the representation of one’s real body, a less integrative metacontrol state should indeed work against the illusion while a more integrative state should support it. As a method to bias participants towards persistence or flexibility, we used two kinds of creativity tasks: the Remote Associates Task (RAT; Mednick, 1962) and the Alternate Uses Task (AUT; Guilford, 1967). Both tasks call for some degree of top-down control (as they require a particular problem to be solved) and search (for the solution of this problem), and thus arguably require some degree of persistence and some degree of flexibility. And yet, the RAT requires the highly constrained search for one possible solution (convergent thinking in the terminology of Guilford, 1967) while the AUT requires a loosely constrained search for as many solutions as possible (divergent thinking)—which implies that the RAT relies more on persistence and less on flexibility than the AUT (Hommel, 2012).

Assuming that this leads people to establish a more persistence-heavy metacontrol state when working on the RAT and a more flexibility-heavy state when working on the AUT, studies have used these tasks to prime metacontrol towards persistence and flexibility, respectively, and to test whether the latter leads to more integration than the former. For instance, Colzato, van den Wildenberg, and Hommel (2013) found evidence of more self–other integration in a joint Simon task when it was interleaved with an AUT than when it was interleaved with an RAT. Along the same lines, Sellaro, Hommel, de Kwaadsteniet, van de Groep, and Colzato (2014) found more interpersonal trust when participants just completed an AUT than when they just completed an RAT. As these observations confirm the assumption that RAT and AUT are effective in biasing people’s metacontrol state towards persistence and flexibility, respectively, we used these two tasks as metacontrol primes in the present study as well. Hence, we not only assumed that performing the RAT and the AUT would lead participants to establish metacontrol states biased towards persistence and flexibility, respectively, but we also assumed that these states would be sufficiently inert to affect the following VHI induction (i.e., the synchrony manipulation) accordingly. To make sure that the induced metacontrol state would still be sufficiently strong during this induction, we interleaved creativity task and (a)synchrony presentation in such a way that participants switched repeatedly between the two.

Taken altogether, we thus predicted that the VHI would be more pronounced if carried out in the context of an AUT than if carried out in the context of an RAT. We further considered the possibility that being exposed to synchrony vs. asynchrony between one’s own and an artificial hand might also have an impact on metacontrol, which in turn might influence performance in the two creativity tasks. In particular, if being exposed to synchrony would bias metacontrol towards flexibility (i.e., integration), it is possible that this would be more beneficial for performance on the AUT than for performance on the RAT, while the opposite would hold for exposure to asynchrony. To test these possibilities, we thus looked into both the impact of engaging in a particular creativity task on VHI (as measured by comparing synchronous with asynchronous conditions) and the impact of experiencing synchronous and asynchronous conditions on performance in the creativity tasks. Our main measure referred to the subjective perception of ownership and agency (Botvinick & Cohen, 1998), but we also included an implicit measure (perceptual drift rates; Kalckert & Ehrsson, 2012) for explorative purposes.

## Method

### Participants

Twenty healthy young adults, with a mean age of 20.1 years (SD = 1.2; range 19–23; 7 male), from Southwest University, China, participated in this experiment for a financial reward. Given the unpredictable effect size, the sample size was chosen following our lab standard for novel manipulations. Participants were naive with respect to RHI/VHI. Written informed consent was obtained from all participants before the experiment. The protocol was approved by the local ethical committee (Southwest University, Faculty of Psychology).

### Experimental setup

**Fig. 1:**
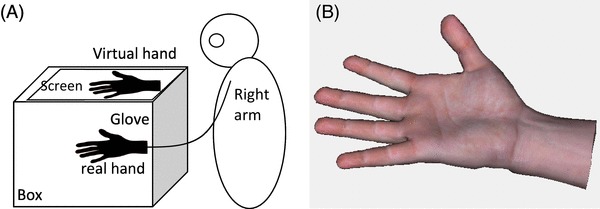
The experimental setup. **a** The participant put his/her real hand inside the box while the virtual hand was shown on the screen placed on top of the box; i.e., the virtual hand appeared on top of the real hand (Ma & Hommel, 2015). **b** The premade 3D virtual hand model

The study was performed in a virtual reality environment. The experimental setup was the same as in an earlier study (Ma & Hommel, 2013; Ma, Lippelt & Hommel, 2017), as shown in Fig. 1a. It is comprised of a data glove (5DT, measurement frequency = 75 Hz, latency = 13 ms) with 14 high-accuracy joint-angle sensors to accurately measure hand and finger real-time motions; a black box (45 cm width × 15 cm height × 35 cm depth) into which the participant put his or her right hand along the depth axis; a cloth which was placed over the participant’s right shoulder to cover the space between the virtual effector and the participant (i.e., participants could not see their real right hand); and the virtual reality software Vizard (http://www.worldviz.com/). We imported a premade 3D virtual hand model (as shown in Fig. 1b) and the data glove module into Vizard, so that the virtual hand received the digital joint-angle data collected with the data glove sensors from the real hand movement of the participant. Given the refresh rate of the equipment, the time delay in all conditions was around 13 ms (on top of the intentional delay in the asynchrony condition).

### Virtual hand illusion

Each VHI condition consisted of an illusion-induction phase of about 120 s, during which participants were to move their real hand freely and could watch the corresponding movements of the virtual hand, either in synchrony or out of synchrony (i.e., with a 3-s delay; see Ma & Hommel, 2013).

### Questionnaire

To assess the extent to which participants experienced the VHI, we used a short version of the standard RHI/VHI questionnaire (Botvinick & Cohen, 1998; Slater et al., 2008; Ma & Hommel, 2015). The statements for virtual hand conditions contained two questions: Q1 (“I can freely control this virtual hand as I wish”) to assess the sense of agency and Q2 (“I felt as if the virtual hand on the screen were my right hand”) to assess the ownership illusion. For each statement, participants responded by choosing a score on a seven-point (1–7) Likert scale, ranging from 1 for ‘strongly disagree’ to 7 for ‘strongly agree’, and 4 means ‘uncertain’.

### Proprioceptive drift

As an implicit measure of the illusion, proprioceptive drift was used—which assesses the degree to which participants feel their real right hand to be located nearer to the virtual hand after the illusion was induced. Participants were asked to use the index finger of their left hand to touch a position on a board, which was attached to the left side of the box, to indicate the felt vertical position of their right index finger (Kalckert & Ehrsson, 2012). Participants pointed before and after each block—with the discrepancy between these two measures being the main dependent variable. During the process, participants kept their right hand flat and static on the bottom of the box. The proprioceptive drift result was then calculated by subtracting the post-block position from pre-block position. With the screen level set to be zero, positive values indicate that participants exhibited an upward drift of the real hand position, i.e., a drift towards the virtual hand.

### Remote associates task (RAT)

In the RAT (Mednick, 1962), participants are presented with three words and are asked to find a common associate (e.g., cottage, swiss, and cake = cheese). We used six Chinese RAT versions, each containing five different items. Each version was to be responded to within 2 min.

### Alternate uses task (AUT)

In the AUT (Guilford, 1967), participants are presented with a common household item and are asked to list as many possible uses as they can within a particular time limit. We used six items (pen, newspaper, tower, bottle, brick, and paper clip, all in Chinese) and had participants respond within 2 min for each one. As usual, the results were scored according to fluency, flexibility, originality, and elaboration. We will report all data but will focus on flexibility (the number of responses, weighted by the number of different categories), the theoretically most transparent measure and the one that in our research turned out to be the most consistent and reliable indicator of divergent thinking (e.g., Akbari Chermahini & Hommel, 2012; Colzato, van den Wildenberg, & Hommel, 2013).

### Design and procedure

A within-participant design was used. Each participant came to the lab to be tested twice (i.e., for two sessions); the synchrony and the creativity task (RAT or AUT) were both (fully counterbalanced) within-participant factors. Half of the participants were tested with RAT in the first session and AUT in the second session, the other half were tested in the reverse order. Half of the participants always experienced the synchronous followed by the asynchronous VHI condition in each session, while the other half always experienced the reverse order. Following the protocol of Colzato et al. (2013), we interleaved the VHI (synchronous or asynchronous) manipulations with AUT items/RAT versions, so to increase the possibility that creativity task-induced metacontrol states would affect the VHI assessment. The experiment was thus composed of 12 (six synchronous and six asynchronous) VHI manipulations, six RATs, and six AUTs in total.

Every time a participant came to the lab, he or she was seated in front of the black box with a computer monitor on its top. The participant would wear the data glove on his or her right hand, and then put his or her hand inside the box, would take on the cloth, and look down on the monitor, where the creativity tasks and the VHI conditions were presented. During the VHI induction, the virtual hand was visible on the monitor while the creativity task material was invisible; and the opposite was true during the creativity task. Participants replied to the questionnaire and the creativity tasks verbally, and their answers were recorded by the experimenter.

Table 1 shows an example of the experimental procedure (i.e., one of the four balanced combinations of creativity task and synchrony). Each of the two sessions consisted of two blocks. In the first block, the participant would first undergo the first perceptual drift measurement and then being presented with one kind of creativity task (e.g., RAT). He or she would then switch between performing the three versions of this task and the three synchrony manipulations of the same type (e.g., synchronous motion of own and virtual hand). Performing the entire block thus resulted in having completed three versions of the same creativity task and three versions of the same synchrony condition. Finally, the participant would respond to the two questionnaire items and undergo the second perceptual drift measurement to assess perceived agency and ownership. In the second block of the same session, the creativity task would stay the same (RAT in this example), but three new versions would be presented, and the synchrony condition would differ (asynchronous motion of own and virtual hand in the example). The participant would again undergo the first perceptual drift measurement, switch between three versions of the creativity task and three new synchrony manipulations. Finally, the participant would again undergo the second perceptual drift measurement and respond to the agency and the ownership item. Performing the entire session thus resulted in having completed six versions of the same creativity task, three versions of one of the two synchrony conditions and three versions of the other synchrony condition. The second session would have the same structure (including the order of the synchrony condition), only that the creativity task would change. Briefly, participants were asked to constantly switch between performing the AUT/RAT for 2 min and experience VHI for 2 min. In total, participants were to switch between the creativity tasks and the VHI manipulations for three times in each block. Between each two blocks, but not within every block, participants were asked to take a 2 min rest to reduce the possible bias from previous block.

**Table 1:** The experimental design and procedure

First session	Block 1	PD1, RAT, VHI(sync), RAT, VHI(sync), RAT, VHI(sync), PD2, Q
	Block 2	PD1, RAT, VHI(async), RAT, VHI(async), RAT, VHI(async), PD2, Q
Second session	Block 1	PD1, AUT, VHI(sync), AUT, VHI(sync), AUT, VHI(sync), PD2, Q
	Block 2	PD1, AUT, VHI(async), AUT, VHI(async), AUT, VHI(async), PD2, Q

*PD1* pre-block proprioceptive drift, *PD2* post-block proprioceptive drift, *Q* questionnaire, *sync* synchronous, *async* asynchronous

## Results

A significance level of *p* < 0.05 was adopted for all tests.

### VHI

**Fig. 2:**
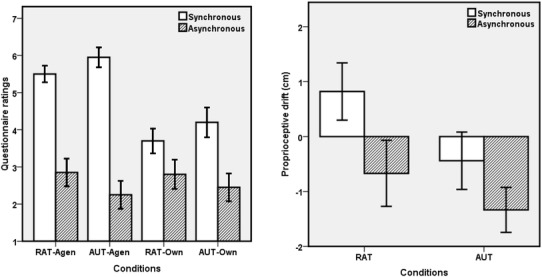
Left panel: Questionnaire scores for agency and ownership, as a function of creativity task and synchrony. Agen: agency, Own: ownership; Right panel: Proprioceptive drift results as a function of creativity task and synchrony. Error bars represent ± 1 standard error

We submitted the agency and ownership ratings to a 2 (task: RAT vs. AUT) × 2 (synchrony: synchronous vs. asynchronous) repeated-measures ANOVA (in which the synchrony effect indicates the illusion) separately. As indicated by Fig. 2 (left panel), the main effect of synchrony was significant for both agency, *F*(1,19) = 69.47, *p* < 0.001, *ηp*2 = 0.79; and ownership, *F*(1,19) = 28.83, *p* < 0.001, *η*p2 = 0.60. While the task did not yield any effect, *F*s(1,19) < 1, the interaction was significant for both agency, *F*(1,19) = 5.44, *p* = 0.031, *ηp*2 = 0.22; and ownership, *F*(1,19) = 5.03, *p* = 0.037, *ηp*2 = 0.21. For both items, the interaction was due to a smaller synchrony effect in the context of the RAT than in the context of the AUT: two-tailed paired t-tests of the synchrony effect (score_synchronous_ − score_asynchronous_, which represents the strength of the VHI) in the RAT and AUT conditions yielded significant differences for agency (2.65 vs. 3.70), *t*(19) = 2.33, *p* = 0.031, *d* = 0.53, and ownership (0.9 vs. 1.75), *t*(19) = 2.24, *p* = 0.037, *d* = 0.61.

We also submitted the proprioceptive drift results to a 2 (task: RAT vs. AUT) × 2 (synchrony: synchronous vs. asynchronous) repeated-measures ANOVA (in which the synchrony effect indicates the illusion). As indicated by Fig. 2 (right panel), the main effect of synchrony was significant, *F*(1,19) = 4.79, *p* = 0.041, *ηp*2 = 0.20, with the drift ratings significantly higher in synchronous (mean = 0.19, standard error = 0.38) than in asynchronous (mean = − 1.00, SE = 0.30) conditions. Neither the task effect nor the interaction yielded any effect, *F*s(1,19) < 3.13, *p*s > 0.093.

### Creativity task performance

Of the three creativity measures per block, we considered the first (which preceded all synchrony manipulations) to represent the baseline and the average of the second and third to reflect effects of synchrony on creativity. The changes from baseline (i.e., average of second and third measure minus first measure) were submitted to a 2(task: RAT vs. AUT) × 2 (synchrony: synchronous vs. asynchronous) repeated-measures ANOVA. In the following, we present the findings for RAT and AUT-flexibility (for other AUT scores, see “Appendix”).

**Fig. 3:**
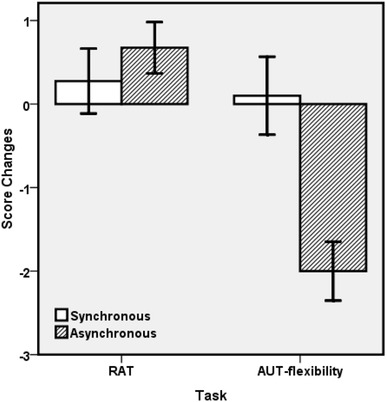
Creativity task scores for RAT and AUT (flexibility) score changes from baseline in all four blocks. Error bars represent ± 1 standard error

As indicated by Fig. 3, the main effect of synchrony was significant, *F*(1,19) = 8.12, *p* = 0.010, *ηp*2 = 0.30, as was the main effect of task, *F*(1,19) = 10.70, *p* = 0.004, *ηp*2 = 0.36. Most importantly, the interaction was also significant, *F*(1,19) = 8.48, *p* = 0.009, *ηp*2 = 0.31. Separately, hypothesis-driven two-tailed paired t-tests yielded no significant differences between score changes for RAT and AUT in synchronous conditions, *t*(1,19) < 0.3, while there was a significant difference in asynchronous conditions, *t*(19) = 6.81, *p* < 0.001, *d* = 1.81: as shown in Fig. 3, the RAT score increases (0.68, SE = 0.31) while the AUT-flexibility score is reduced (− 2.0, SE = 0.35) in asynchronous conditions. Additional exploratory t-tests, in which we Bonferroni-corrected the significance criterion for multiple comparisons (*α* = 0.05/4 = 0.0125), confirmed that, in the asynchronous conditions the negative-going AUT score changes were significantly different from zero, *t*(19) = 5.66, *p* < 0.001, *d* = 1.26, while the positive-going RAT score changes were not, *t*(19) = 2.20, *p* = 0.04.

## Discussion

The findings are clear-cut. As in previous studies, participants were more likely to experience subjective agency and ownership for a virtual hand if it moved in synchrony with their own, real hand. As predicted, the size of this effect was significantly moderated by the type of creativity task in the context of which the illusion was induced. In the context of the RAT, which we assume to bias metacontrol towards persistence (and thus increase top-down control and event discrimination), the illusion was markedly smaller than in the AUT context, which we assume to bias metacontrol towards flexibility and integration. This suggests that the perception of body ownership in general, and the integration of candidate effectors in particular, are moderated by metacontrol. More specifically, persistence seems to support self–other segregation while flexibility promotes self–other integration. Given that persistence and flexibility was induced by means of tasks without any obvious social or self-related implications, this finding is consistent with TECs assumption that the cognitive representations of body parts are no different from cognitive representations of body-unrelated events and objects. The finding also fits with predictions from MSM, especially with respect to the impact of metacontrol on event integration.

It is interesting to note that the explicit measure of ownership, as assessed by the questionnaire, was sensitive to the metacontrol manipulation while the implicit measure, as assessed by the perceptual drift, was not. Like many previous dissociations between explicit and implicit measures of ownership (Holmes, Snijders, & Spence, 2006), this observation provides converging evidence that the two measures do not rely on exactly the same information. It may be that conscious self-perception of ownership is depending on more, or more integrated information than proprioceptive drift rates, which may rely more on multisensory discrepancy (Rohde, Di Luca, & Ernst, 2011).

The creativity tasks did not only serve to induce metacontrol-biases but they were also affected by the synchrony manipulation, which was also thought to be able to induce different metacontrol states. The outcome pattern seems to suggest that perceiving asynchrony between events promotes convergent, but impairs divergent thinking, while perceiving synchrony has little effect. The reason may be the relatively low illusion strength: the ownership ratings for synchronous conditions were not very high, and the ownership ratings for asynchronous conditions were quite low. This might suggest that synchrony did not induce strong flexibility but asynchrony induced strong persistence. If so, the asynchrony conditions would indeed be expected to improve performance in tasks that rely on persistence, such as the RAT, and to impair performance in tasks that rely on flexibility, such as the AUT.

It is important to keep in mind the fact that our present findings were obtained in a paradigm that strongly interleaved what we considered the task prime (i.e., the particular creativity task) and the induction of the VHI—the process we aimed to prime. The practical reason to do so was to increase the probability that the metacontrol state that the creativity tasks were hypothesized to induce or establish would be sufficiently close in time to the synchrony manipulation to have an impact on the thereby induced changes in self-perception. However, this implies that we are unable to disentangle the effects of the task prime proper and the effects of possible interactions between this task prime and the synchrony manipulation. There are indeed reasons to assume that such interactions are not unlikely to have occurred and that they would make perfect theoretical sense. The observation that the VHI was affected by the type of creativity task and performance in the creativity tasks was affected by the synchrony manipulation suggests some degree of overlap between the ways that engaging in particular creativity tasks and experiencing particular degrees of synchrony are able to bias perceived ownership and agency. In terms of our theoretical framework, this implies that engaging in divergent thinking biases metacontrol towards flexibility in similar ways as experiencing synchrony between one’s own movements and those of a virtual effector does, while engaging in convergent thinking biases metacontrol towards persistence as experiencing asynchrony does. What the present findings demonstrate is that both kinds of manipulation together bias the VHI in the predicted direction, but they do not allow to statistically or numerically separate and estimate the contribution that each of the two confounded manipulations might have made. Accordingly, the present findings should not be taken to provide conclusive evidence that priming tasks alone are able to change self-perception without being supported (and perhaps even enabled) by the experience of synchrony between proprioceptive and visual action feedback.

## Appendix

AUT scores (means and standard errors of change from baseline) as a function of synchrony.

**Table:**

	Synchronous	Asynchronous
AUT-fluency	− 0.33/0.48	− 2.25/0.48
AUT-elaboration	− 0.23/0.19	0.15/0.21
AUT-originality	1.00/0.22	− 0.68/0.37
